# The Necessity of a Library

**Published:** 1920-10

**Authors:** D. M. Cattell

**Affiliations:** Nashville, Tenn.


### THE NECESSITY OF A LIBRARY

D. M. CATTELL, A.M., D.D.S., NASHVILLE, TENN.

In this day but little new is told, yet many different expressions are uttered, each new thought is turned and twisted about until the sound of these utterances seems so different from that heard before that we are apt to regard it as new. When the time comes for us to express our own thought, we wonder what has been said before and who spoke it. Only large libraries have the data. To write intelligently, the would-be author must consult the records, so, in expressing himself, he will not repeat former writers, except to give credit to whom it is due. If one should express a new thought or give a new version of known facts, he wants to go on record as such author, and that record should be so placed that all others may see, and be guided accordingly.

No individual can maintain such a library of dental journals as to cover the whole field of monthly or quarterly publications, hence the burden must be carried by either national, state or local societies, or by dental educational institutions. As the former organizations have no fixed abode, it necessarily falls to the lot of the dental schools to gather copies of all dental publications, be they annual, semi-annual, quarterly, monthly or bimonthly. Also, copies of all dental books published as text-books for students or general reading for the graduate profession. All these should be so arranged that one wishing to consult any author can readily find the article wanted, and, when found, there should be opportunity to sit and read and copy, if necessary. This necessitates a large and airy room, lined with shelves filled with complete volumes of all dental literature, dictionaries, lexicons and encyclopedias, and chairs and tables so arranged as to be utilized. There should also be copies of standard charts; in fact, every record available for study or reference. It necessitates a librarian who knows, in a general way, the literature of the profession, and so in love with it that the searching for certain articles wanted is a pleasure only satisfied when the required information is found.

It so happens that shortly there will be issued through the "American Institute of Dental Teachers" an index of all articles published since the publication of the first dental journal in America, covering a period from about 1850 to the present time. This complete index covers the ground from three angles.

1. The articles are enumerated under certain heads, as operative dentistry, dental pathology, histology, etc.

2. The articles are tabulated under their special titles, as non-cohesive gold fillings, the faces of pluggers, inflammation of the dental pulp, etc.

3. All papers, discussions, books written and published are given under the writer's name.

With such help before us, any one can soon learn just what an individual has written or said on any professional subject, anywhere at any time, provided such utterance was published in some dental journal or text-book. Considering these, a searcher for what has already been written on any dental subject can soon find every thought published. This index volume will soon be in every well-equipped dental library. Subsequent volumes will be added every five years or so.

*Vanderbilt University, School of Dentistry,* has undertaken to establish such a library. She has already gathered together some eight hundred bound volumes and many volumes of unbound dental journals and thousands of miscellaneous pamphlets and proceedings of special and state organizations. There are many incomplete volumes that can only be made complete through the interest of her friends and alumni. We are asking all such to send us their journals, of any and all titles and dates. Those not needed can be readily exchanged for needed ones with other dental libraries.

*The Journal of the National Dental Association* is acting as a clearing-house, so to speak, for the exchange of dental journals. There is also an organization under the title of ''American Library and Museum Association,'' with headquarters at present with the First District Dental Society of New York, Dr. A. F. Ishman, president, and Dr. B. W. Weinberger, secretary. This organization also proposes to act as a medium of exchange.

*The Northwestern University, School of Dentistry,* also has many duplicate copies which the librarian will exchange for copies wanted to complete certain files. Hence all extra copies can be exchanged for those needed.

*Vanderbilt Library* is anxious to get dental college announcements, bulletins and catalogs; also those of post-graduate courses; indeed, *anything pertaining to dentistry.*

We are expecting to open soon a reading and study room for students, teachers and professional men from anywhere. To accomplish our aim, we ask any who have the interest of accomplishment at heart to send us all printed dental matter they feel willing to spare. In return we promise to put to its best use all intrusted to our care. More than that, if any one has a private library, dental or otherwise, and will send it to us, it shall be put in a case and labeled with the donor's name as well as each individual book or volume.

''There is a crying need of improved dentistry, and one of the principal means of advancement is the betterment of its literature. To do this, however, necessitates *dental libraries.* Now is your opportunity to aid in the building and strengthening, as well as preserving all the old files and the interesting historical material.''

(The above is a copy of a paragraph taken from a recent letter from the secretary of the ''American Dental Library and Museum Association,'' and *Vanderbilt* has applied to for membership in the organizatioin.)

In another article for the next *Quarterly Journal* I want to tell about provision being made for a museum. In the meantime we are receiving specimens of antique instruments, tooth anamolies, pathologic specimens and skulls of man and beast, miscellaneous specimens of all kinds of interest to the student of nature and the searcher after truth. The later specimens coming in are a lion's skull and a deep-sea turtle.

Just now we particularly want one of those dental "chests" with the instruments inlaid, so to speak (each fitting into its own arranged notch). The mirror handles of mother of pearl much carved and handles of instruments of ivory, onyx or pearl. Vanderbilt Dental Museum will never rest easy until one of these chests can be seen within its doors. Who will help us to locate one? And who will help us to build the library and museum to such proportions that the South will be proud of it? Standing, as it does, for education, for progress and for higher attainment.

Nashville is situated centrally in the state; more often than elsewhere our state organization meets therein. It could be made a great rendezvous for dentists throughout the South in their search for data, for facts and for truth.

*"The library and museum of an institution should be the hub around which practically all the activities of that institution should revolve.*

"There should be a teaching connection between the library and museum and every department of the institution. The library should be the place where the student will be attracted when he has no other definite assignment. It should be made so attractive that he will naturally drift there when he has leisure time."

A *library* has been classified by Dr. Hoff of the University of Michigan as follows: (1) Historic literature; (2) scientific research; (3) published books; (4) current literature; ((5) portraits and history of eminent men of the profession.

A museum might be classified as follows: (1) Antique instruments and appliances of dentistry; (2) anatomical and pathological specimens; (3) comparative anatomy specimens; (4) instructive material for classwork; (5) technical material (students' product in technical laboratories).—*Tennessee State Dental Journal.*

